# Negotiating new roles in general practice: a qualitative study of clinical pharmacists

**DOI:** 10.3399/BJGP.2023.0145

**Published:** 2023-12-29

**Authors:** Fay Bradley, Pauline A Nelson, Chris Cutts, Damian Hodgson

**Affiliations:** School of Health Sciences, Faculty of Biology, Medicine and Health, University of Manchester, Manchester.; School of Health Sciences, Faculty of Biology, Medicine and Health, University of Manchester, Manchester; Sheffield University Management School, University of Sheffield, Sheffield.; Centre for Pharmacy Postgraduate Education, Division of Pharmacy and Optometry, Faculty of Biology, Medicine and Health, University of Manchester, Manchester; NHS England North West, Manchester.; Alliance Manchester Business School, University of Manchester, Manchester; Sheffield University Management School, University of Sheffield, Sheffield.

**Keywords:** general practice, pharmacists, professional role, workforce, qualitative research, clinical skills

## Abstract

**Background:**

To address general practice workforce shortages, policy in England has supported the recruitment of ‘non-medical’ roles through reimbursement funding. As one of the first to receive funding, the clinical pharmacist role offers insight into the process of new role negotiation at general practice level.

**Aim:**

To identify factors influencing clinical pharmacist role negotiation at practice level, comparing the process under two different funding and employment models.

**Design and setting:**

Qualitative interview study with staff involved in the following schemes: 1) the national NHS England (NHSE) Clinical Pharmacists in General Practice scheme; and 2) a local clinical commissioning group-funded scheme, providing clinical pharmacist support to general practices in one area of Greater Manchester in the UK.

**Method:**

Semi-structured interviews with purposive and snowball sampling of pharmacists, GPs, and practice staff took place. The interviews were analysed using template analysis.

**Results:**

In total, 41 interviews were conducted. The following four factors were found to influence role negotiation: role ambiguity; competing demands and priorities; potential for (in)appropriate utilisation of clinical skills; and level of general practice control over the role. Key differences between the two funding and employment models were the level of influence GPs had in shaping the role and how adaptable pharmacists could be to practice needs. The potential for inappropriate utilisation was reported under both schemes, but most apparent under the role reimbursement, direct employment model of the NHSE scheme.

**Conclusion:**

This study has highlighted lessons applicable for the introduction of non-medical roles more widely in general practice. It has provided insight into the factors that can influence role negotiation at practice level and how different funding and/or employment models can impact on this process.

## Introduction

Developing and expanding the ‘non-medical’ workforce to support and sustain general practice, in the face of decreasing GP numbers and increased workload, has been a key policy initiative for the NHS in England since 2014.^1^^,^^2^ This has led to the development of a range of new roles in general practice, including paramedics, physician associates, social prescribers, first-contact physiotherapists, and clinical pharmacists. The deployment of pharmacists into general practice has been central to these plans and, in 2015, NHS England (NHSE) provided a large injection of national funding under the Clinical Pharmacists in General Practice (CPGP) scheme to boost employment.^3^ General practices in Scotland and Northern Ireland have since seen additional investment for the employment of pharmacists. The movement of pharmacists into general practice roles has also gained momentum elsewhere, including Australia, New Zealand, and Canada.^4^^–^^6^

General practice clinical pharmacists are non-dispensing pharmacists, who can undertake a range of tasks, including medication reviews, medicines optimisation, and running clinics with patients. They may also be qualified independent prescribers. In England, pharmacists working in general practice tend to be either directly employed by a practice, work for a primary care network (PCN), or operate under an alternative model such as in locality-based pharmacy teams working across practices. When the NHSE CPGP scheme pilot launched (phase 1, 2015–2017), practices in areas with GP shortages were prioritised. For phase 2 (2017–2020), demonstrable ‘working at scale’ was emphasised, meaning pharmacists were more likely to work across ≥2 practices. Tapered 3-year funding was provided, with practices required to cover the full cost from year 4 if they decided to retain the pharmacist. A number of conditions were attached to the funding offer, namely that the pharmacist would: be embedded in the general practice team; work in a patient-facing role; undertake an 18-month training programme; and complete an independent prescriber qualification (if not already a prescriber). Funding ran until March 2020 and was then consolidated into the Network Contract Directed Enhanced Service: Additional Roles Reimbursement Scheme (ARRS).^7^

**Table:** How this fits in

Studies on the introduction of clinical pharmacists into general practice have highlighted general challenges related to role definition and clarity, but little is known about how roles are negotiated and defined at local practice level. This study identified four factors influencing the negotiation of the clinical pharmacist role and has demonstrated how negotiation may be affected by the funding and/or employment model. These findings have wider applicability for the introduction of other non-medical roles into general practice.

An increased focus on ‘at-scale’ working also led to the concurrent development of teams of pharmacists working with and across general practices at a locality level. Pharmacists working in these teams were often employed by a provider organisation, such as an NHS hospital trust or GP-led provider organisation, to offer a general practice pharmacy service to a particular geographical area. Funding for these initiatives came from a variety of sources such as the local clinical commissioning group (CCG) or the aforementioned NHSE CPGP scheme.

The redeployment of pharmacists into general practice roles is often considered to be one of the success stories of NHSE’s primary care workforce reforms, demonstrated through recurrent and increased national funding, despite limited evidence of tangible benefit and impact.^8^^–^^10^ As one of the first ‘new’ general practice roles to be targeted for national reimbursement funding, pharmacists could be viewed as early pioneers and their experiences may have wider applicability to the introduction of other new non-medical roles. Previous research, drawing on small, localised studies of pharmacist trainees and directly employed (non-NHSE scheme) pharmacists, has suggested that the clinical pharmacist role lacks clarity,^11^ which can lead to interprofessional tension.^12^

In practice, role definition may evolve depending on the pharmacist’s experience level, competency, professional interest, and general practices’ individual priorities and needs.^13^ The current literature, however, does not consider how the role may also be influenced by conditions related to the employment and/or funding model. Therefore, clinical pharmacists and general practice staff may enter a process of role negotiation, influenced by a series of external and internal contextual factors, which affect how each pharmacist embeds into the practice and ultimately contributes to patient care.

In this article, the experiences of general practice pharmacists, GPs, and other staff are described as they negotiate and define the practice pharmacist role. The experiences are examined and compared under two different funding and employment models: 1) national NHSE CPGP scheme-funded practice-employed pharmacists; and 2) local CCG-commissioned, NHS trust-employed pharmacist teams, providing clinical pharmacy services to practices in one Greater Manchester locality. Box 1 outlines the key features of these two models, referred to henceforth as ‘NHSE’ scheme and ‘Locality’ scheme. The study also discusses lessons of relevance for the ongoing introduction of other non-medical roles into general practice.^14^

**Box 1.** Key features of the NHS England and Locality schemes

	**NHS England scheme**	**Locality scheme**
**Funder**	NHS England Clinical Pharmacists in General Practice programme (tapered role reimbursement funding to practices for first 3 years)	Clinical commissioning group
**Employment model**	Direct employment by general practice	External employment by NHS trust
**Work model**	Practice-based individuals (mostly working at one practice but may work across practices)	Place-based teams (working across practices on sessional basis)

## Method

To explore experiences and perceptions of negotiating and defining the practice pharmacist role, a qualitative study design was adopted. Qualitative interview data for both schemes were captured through two discrete studies: 1) data on the NHSE scheme were drawn from a national evaluation of the NHSE CPGP scheme phase 1 mandatory training programme; and 2) data on the Locality scheme were drawn from a process evaluation of the introduction of new roles into general practice, which included locality pharmacist teams, in one Greater Manchester area.

The national evaluation of the NHSE CPGP scheme phase 1 training programme was a mixed-method evaluation; the quantitative longitudinal element of the study has been reported elsewhere.^15^ This article has drawn on the qualitative element, which involved semi-structured interviews with phase 1 pharmacists and GPs acting as clinical supervisor (each NHSE scheme pharmacist was assigned a GP clinical supervisor). A message asking for expressions of interest to take part and the research team’s contact details was sent to all phase 1 pharmacists (*n* = 457) via the training programme’s online platform. Recruitment of GP clinical supervisors was via phase 1 pharmacists and their education supervisors, using snowball sampling. All interviews were conducted by one author by telephone, and participants provided verbal informed consent. GP clinical supervisor interviews were conducted mid-training (March–May 2017) and pharmacist interviews at the end of the training programme (February–March 2018).

For the introduction of new roles into general practice process evaluation, which included locality pharmacist teams, participants were sampled purposively on their professional role. Locality scheme leads were identified first and invited to interview. Locality team pharmacists and GP practice staff from host practices were identified through snowball sampling via scheme leads and invited to interview. At the start of interviews, the Locality team included 22 pharmacists. Interviews were conducted face to face by two authors between May 2017 and May 2018, and participants provided written informed consent.

Interview topic guides (see Supplementary Information S1) based on the aims of each evaluation and previous work and literature were developed and piloted; conversation was also allowed to develop naturally to discuss other aspects relevant to the role. Interviews lasted between 21 and 70 minutes. All interviews were audiorecorded with participants’ permission, transcribed verbatim by a University of Manchester-approved transcription company, checked for accuracy by two authors (one for the NHSE scheme study and one for the Locality scheme study), and anonymised.

In both studies, data collection closed once the research team judged that data categories were sufficiently well developed to meet the study aims. Preliminary coding of transcripts was undertaken independently by one author (NHSE scheme study) and three authors (Locality scheme study), assisted by NVivo (version 11). Codes were categorised, discussed among the team, refined, and grouped into themes. Template analysis^16^ enabled comparison of participant perspectives from different organisational contexts. The analytical focus was on identifying how decisions about the pharmacist’s role and the work they performed were made and negotiated. Data management plans were in place to ensure data security.

## Results

In total, 41 interviews were conducted (*n* = 25 NHSE scheme; *n* = 16 Locality scheme). For the NHSE scheme, 13 pharmacists participated, six of whom were appointed at senior clinical pharmacist level, meaning that they had advanced clinical experience and would support less experienced pharmacists on the NHSE scheme. Twelve GPs acting as clinical supervisors to pharmacists on the scheme also took part. For the Locality scheme, in addition to scheme pharmacists (*n* = 4) and GPs from practices hosting them (*n* = 3), two directly employed practice pharmacists (not funded through the locality or NHSE scheme) and four practice managers took part, along with three Locality scheme leads (a GP lead, provider organisation lead, and CCG lead). Table 1 details participants by role from both studies.

**Table 1.** Participants by role

**Scheme and participant role**	***n***
**NHS England scheme**	
GP clinical supervisors	12
Senior clinical pharmacists	6
Clinical pharmacists	7
**Locality scheme**	
Locality scheme pharmacists	4
Locality scheme leads (GP, clinical commissioning group, and provider organisation)	3
*Practice staff:*	
Practice pharmacists (directly employed, not locality or NHS England scheme funded)	2
Practice managers	4
GPs	3
**Total**	41

The following four factors were found to influence role negotiation: role ambiguity; competing demands and priorities; potential for (in)appropriate utilisation of clinical skills; and level of general practice control over the role.

### Role ambiguity: deciding on the right role

In both schemes, there were reports of a lack of understanding about what the role should entail. GPs in the NHSE scheme described the experience of trying to establish the right role for pharmacists as a lengthy process, with stops and starts, revisions, and renegotiation. The need for ongoing negotiation stemmed from a high level of role ambiguity, which was acknowledged by both pharmacists and GPs. Pharmacists indicated that the process was a struggle, hindered by the role being, *‘a blank canvas – no one really knows what they want to do with you … ’* (NHSE scheme pharmacist 12)*.* Even when agreement had been reached, doubts over whether the model adopted was right for the practice remained:

*‘… I still don’t know yet that we’ve got the right role and responsibility … we want to make the best clinical use of their time, but also keep the pharmacist interested and motivated … there are so many needs within the practice that the pharmacist could input into and yet they only have so many hours in the day like the rest of us.’*

(NHSE scheme GP 7)

Most GPs interviewed from the NHSE scheme expressed desire for further support, information, or guidance on work planning and utilisation of the pharmacist’s skills. NHSE scheme pharmacists felt that there was a need for more shared knowledge and experiences to promote the patient-facing aspect of the role to GPs and other practice staff:

*‘There needs to be more case studies … not necessarily the stuff on paper because the GP’s not going to read it … There needs to be like GPs saying … our pharmacist was fantastic and this is what we got them to do … we’re now working very well and they … can be an integrated member of the team.’*

(NHSE scheme pharmacist 4)

Locality scheme pharmacists described their role and tasks performed as similar to those of NHSE scheme pharmacists. However, not being directly employed by the practice and providing a service commissioned by the local CCG reportedly led to some confusion about whether they should be carrying out CCG pharmacist roles:

*‘… it’s a really weird situation … the practice manager came to me and said “Oh …* [the] *CCG pharmacist has said that you can do this piece of work.” And I was like oh hang on really not something that we’ve been told was a priority, very much the work that the CCG pharmacists normally do … And for me to turn around and say, “that’s not really my job* [and] *I haven’t got the time to do it because I’ve got all this other stuff to do”, feels uncomfortable.’*

(Locality scheme pharmacist 1)

Although, like the NHSE scheme, there was still confusion at practice level about what the role entailed, the Locality scheme was said to be more focused on specific tasks and priorities than the NHSE scheme. This resulted in less negotiation at practice level between individual GPs and pharmacists to decide on the ‘right role’, as the Locality pharmacists’ work was decided on and planned by their employing organisation rather than by the practice:

*‘We’re very much influencing, deciding … what the balance is going to be regards work, and I think it will be slightly different in every practice.’*

(Locality scheme provider lead)

Overall, role ambiguity was greater for the NHSE pharmacists, being a new scheme with limited guidance. The Locality scheme was more focused on particular tasks, as defined externally by the provider organisation; however, role confusion and ambiguity persisted at practice level.

### Competing demands and priorities

Both schemes came with competing demands, priorities, and expectations, which pharmacists were struggling to reconcile. Locality pharmacists described the pressure and difficulties they faced trying to balance three different and potentially conflicting agendas: the provider organisation’s (employer) aim to standardise and improve quality of care across the locality’s practices; the general practice’s (host) aim to ease GP workload; and the CCG’s (commissioner) aim to reduce costs. Pharmacists described how they had to handle these different agendas *‘sensitively’* (Locality scheme pharmacist 1), showing willingness to the practice to embed themselves, while also trying to work towards the other objectives of the service.

Parallel experiences were described by the NHSE scheme pharmacists, who spoke of being *‘pulled in two directions’* (NHSE scheme pharmacist 10), trying to meet the practice’s need to relieve GP workload by carrying out clinical administration (back-office) work, while also trying to satisfy the demands of the NHSE scheme, which required mandatory patient-facing experience accompanied by dedicated clinical supervision time with a GP. Pharmacists reported that they had to compromise about the type of work they conducted to embed themselves into the practice and protect their job security; under the scheme, after year 3, practices had to decide whether to retain and fund the pharmacist from their own budget:

*‘I think* [the biggest challenge is] *probably balancing that expectation from the practice, from the pharmacists, from* [the training provider] *and being able to support the team sufficiently; I think a lot of* [the pharmacists in the area] *feel a pressure that, you know, can they do enough in the time they’ve got* [left on the scheme] *for a practice not to pull out?’*

(NHSE scheme pharmacist 13)

Although the schemes focused on different priorities, pharmacists on both schemes were facing similar experiences trying to reconcile competing agendas while trying to avoid creating tension to embed themselves in the practice and/or protect their job security.

### Potential for (in)appropriate utilisation of clinical skills

Role ambiguity and the high-level need to relieve GP workload had the potential to lead to inappropriate utilisation of pharmacists’ skills in both schemes. The greatest need for practices tended to be administrative, back-office, non-patient-facing type work (for example, medication queries, medicines reconciliation, and prescription processing) and much of the negotiation process centred on how much patient-facing (clinical) versus back-office (administrative) time the pharmacist’s role should entail. One NHSE scheme GP explained that, having initially defined the role around the pharmacist’s area of interest (patient consultations) and the NHSE scheme training requirements for patient-facing time, it became apparent that this was not a cost-effective use of the pharmacist’s time and did not address the practice’s primary need. This led to a renegotiation of the role and a resultant move to more administrative work dealing with prescription requests:

*‘…* [they] *really enjoyed seeing patients in the surgery but then … we realised that wasn’t a cost-effective use of* [their] *time to us. So, we had a discussion then about … doing more … prescription requests, and less of the face-to-face contact … So, that was a potential area for discord. But I think that was what was required, that was the reason we got a pharmacist.’*

(NHSE scheme GP 10)

Although both schemes faced the potential for inappropriate utilisation of skills, Locality scheme pharmacists reported feeling somewhat protected by their employer (the provider organisation) that *‘… understand what clinical pharmacists can do’* and are *‘directing* [the] *role to make sure* [it is] *clinical’* (Locality scheme pharmacist 1)*.* Locality pharmacists did, however, describe instances where they felt uncomfortable saying ‘no’ when asked by general practice staff to carry out particular tasks that were not part of their role. Locality scheme leads explained that some pharmacists in the team needed support to assert themselves in this respect to prevent inappropriate utilisation:

*‘We did have stories of some* [GPs] *saying, “Great, you’re here, fantastic, there’s today’s re-auth*[orisation]*s, there’s today’s letters, off you go.” And that wasn’t what we were wanting and we had to empower some pharmacists to say, “No, that’s not why I’m here. And I’m very happy to help, but … we can’t just sort of throw them into a practice blindly and half expect them to do whatever the practice throws their way.’*

(Locality scheme GP lead)

NHSE pharmacists also described the difficulties they faced having honest and open dialogue with GPs about utilising other aspects of the role, considering the pressing need to relieve GPs’ workload and being aware of the positive and recognised contribution they could make to ease this pressure:

*‘… is it bad then that you do the paperwork if it eases their time and makes them less stressed? … I was doing a lot of discharges and the GP was like, “Wow, you’ve really helped” … and it feels like you can’t say, “well, I shouldn’t really be doing this”, when they’ve been so ecstatic that you’ve helped them.’*

(NHSE scheme pharmacist 4)

Some GPs were aware of this tension. They were clear that the greatest need within their practices that the pharmacist could fill related to administration, but appreciated that the associated lack of variety and potential underutilisation of clinical skill could be professionally unsatisfying:

*‘In an ideal world, we’d have somebody who came in and did all of our prescriptions — that would be fab. We could then live without* [them] *doing anything else, but obviously it’s a job that has to be satisfactory for* [them] *as well.’*

(NHSE scheme GP 12)

*‘… just doing the discharge summaries, initially* [a] *poor* [pharmacist] *got totally swamped with them and* [they] *could just do nothing else and do those; but obviously there’s other areas that* [they’ve] *got to concentrate on.’*

(NHSE scheme GP 6)

There was recognition among interviewees from both schemes that, over time, the inclusion of pharmacy technicians into the general practice team or the upskilling of existing administrative staff could aid more appropriate utilisation of practice pharmacists, releasing them from some of the back-office administrative work and facilitating a greater patient-facing clinical role:

*‘*[I] *probably do a bit too many simple reviews rather than as many complicated reviews as I can. For that to change somebody else needs to be trained to do the simple reviews … We do have prescription clerks and the lead of them I think could be trained. A pharmacy tech would be ideal.’*

(NHSE scheme pharmacist 9)

*‘I think quite a lot of the work that our pharmacists do at the moment is almost transcribing … if the pharmacy technician could do a lot of* [that] *… that would save a lot of time for the pharmacists.’*

(Locality scheme GP lead)

In both schemes there was concern about pharmacists’ roles being dominated by administrative tasks and clinical skills being underutilised. The potential for inappropriate pharmacist utilisation was, however, more apparent with the NHSE scheme, being a role reimbursement, direct employment model.

### Level of general practice control

A key difference between the schemes and employment models was the degree of control and negotiating power that the general practice had to influence, shape, and define the pharmacist’s role. In the direct employment model of the NHSE scheme, practices had the greatest control over the pharmacist’s work. However, negotiation was required to meet the conditions of the scheme and training programme (as described above), which curtailed the flexibility of pharmacists to adapt to specific practice need. One GP supervising a pharmacist previously directly employed at the practice, then re-employed through the NHSE scheme, felt that movement onto the NHSE scheme had resulted in a loss of practice control over the direction of the pharmacist’s role. To accommodate the scheme’s requirement for the pharmacist to gain patient-facing experience, some of the non-patient-facing work, previously performed by the pharmacist, had been shifted back to GPs. This GP felt that direct employment outside of a funding scheme allowed the practice full control of the role, to ensure that it was tailored to the individual needs of the practice:

*‘Some of that workload had to go back to GPs to allow us capacity for pharmacists to take on the other roles that the practice were not particularly wanting but the* [NHSE scheme] *wants … and the pharmacists want … Before, the role had grown into what we needed, and of course now it’s not really like that … it’s more about satisfying the requirements of the* [NHSE scheme].*’*

(NHSE scheme GP 8)

As the work direction was set by an external provider, practices involved in the Locality scheme had the least influence over the pharmacist’s work. Some Locality scheme pharmacists felt that this had led to suspicions that they were *‘external audit people … keeping an eye if they are doing the wrong thing or not’* (Locality scheme pharmacist 4). Scheme leads, recognising that practices valued flexibility in the role, had attempted to provide practices with a menu of pharmacist work options for them to choose from. However, GPs reported feeling detached from the service and frustrated by the scheme’s rigidity. They contrasted the level of control they had over the Locality scheme pharmacists’ work with that of directly employed pharmacists:

*‘We haven’t been able to influence* [the Locality pharmacists’] *workload, I’ll be honest with you, whereas the* [directly employed pharmacist] *working for us, we tell her exactly what we want to do, and she’ll go and do it. So that’s a difference.’*

(Locality scheme GP 2)

For some Locality scheme pharmacists, a direct employment model was considered less likely to cause tension and facilitate greater integration. For others it was problematic owing to concern that the pharmacist would have limited power to negotiate their role with the practice, which could lead to inappropriate utilisation. The directly employed pharmacists interviewed for the Locality scheme described ambiguity over their clinical status and a sense that they needed to be available and adaptable to practice demands:

*‘We’re not admin, but we’re not quite full clinical … we are the floaters and the people who are available everywhere.’*

(Directly employed pharmacist 1)

Overall, in both schemes, practices valued having control over the work of the pharmacist and appreciated flexibility and adaptability to practice need. The NHSE scheme provided practices with some but not complete control; the Locality scheme provided the least control for practices.

## Discussion

### Summary

To the authors’ knowledge, this study is the first to identify factors influencing the negotiation and definition of the GP clinical pharmacist role under two different funding and employment models: a national role reimbursement scheme involving direct practice employment; and a locality CCG-commissioned scheme providing practice pharmacy services via externally employed pharmacists. The challenges that pharmacists faced balancing competing demands and priorities were common to both schemes. Ambiguity over the role and concern about the potential for inappropriate or under-utilisation was evident in both models, but greatest for the NHSE scheme. Key differences were the level of influence GPs had in shaping the role and how adaptable pharmacists could be to practice needs. To some degree, mandatory scheme conditions constrained GP control and pharmacists’ adaptability on the NHSE scheme, but GP influence was found to be minimal under the Locality scheme.

### Strengths and limitations

The qualitative design enabled an in-depth exploration of pharmacists and practice staffs’ perceptions. By comparing data from two discrete studies, the study was able to provide insight into how role negotiations and decisions may be influenced by the funding and/or employment context. Limitations of comparing data from these two studies include differences in the purposive sampling approaches resulting in unmatched samples, and different study designs meaning interview questions, interviewers, and modes of data collection (face to face or telephone) varied. In both studies, interviews were conducted in the first 18 months of these schemes and pharmacist roles will have inevitably developed since. However, these early experiences hold relevance for the ongoing introduction of other non-medical roles in general practice.

### Comparison with existing literature

Emerging evidence has suggested that other roles within the ARRS, including paramedics, link workers, and physiotherapists, may be facing similar challenges to the pharmacists in the present study, including role ambiguity, issues around autonomy, and inappropriate utilisation.^17^^,^^18^ Ultimately, deciding who does what in health care involves local negotiation. Negotiated order theory has been applied to various healthcare settings and teams to explain the continual work involved in the organisation and reorganisation of labour,^19^^,^^20^ and it is recognised that some clinicians will hold greater power in these negotiations than others.^21^ Invariably the negotiations described in this study will be influenced by a historical power imbalance in the GP–pharmacist relationship.^22^ Interactions between GPs and community pharmacists have been found to be limited, indirect, and non-reciprocal, with the pharmacist adopting a cautious and deferential approach towards the GP.^23^ With the majority of phase 1 NHSE scheme pharmacists originating from a community pharmacy background,^24^ a shift from an indirect, deferential style of interaction to a more direct, assertive, and dialogical approach may take some adjustment. A study of general practice pharmacists in Canada has suggested that issues around pharmacist isolation and *‘feeling in the way’* persist but may decrease over time.^6^ Pharmacists in the present study spoke about difficulties broaching the subject of the appropriateness of tasks with GPs, stressing that discussions needed to be handled sensitively to show willingness to embed in the team and/or not threaten job security. This was also linked to the struggles they faced balancing competing agendas. A recent study in Northern Ireland also found that pharmacists not employed by the practice faced some difficulty reconciling competing demands from both employers and practice hosts.^25^

Literature has suggested that clearer GP pharmacist role definition and guidance is required.^11^^,^^12^ This study has not only confirmed that guidance may be beneficial in some cases, but also offered a more nuanced picture. For example, it has indicated that, while dictation from external parties can reduce role ambiguity, it can also undermine the perceived value of the role to the general practice. Previous work has suggested that practices value pharmacists’ adaptability and flexibility.^26^ This study has confirmed this and concluded that practices want the ability to shape and tailor the role to their needs; thus, a certain degree of role ambiguity may be tolerated and even considered beneficial, in some respects.

### Implications for practice

These findings hold relevance for the introduction of other non-medical general practice roles introduced under the ARRS, especially in the context of the multiple agendas and demands faced by PCNs.^27^ In particular, they highlight that an unintended consequence of role reimbursement schemes can be the potential for inappropriate or underutilisation of skills. The variety of roles now included in the ARRS offers general practice and PCNs the opportunity to employ more appropriate skill mix for their needs, for example, PCNs can now employ one pharmacy technician (two if >100 000 patient population). These findings also have applicability to other countries operating or considering similar incentive schemes, such as the Workforce Incentive Program in Australia, which provides funding for allied health professionals, including pharmacists, to work in general practice.^28^ This study reiterates the importance of needs being identified before any workforce redesign, to ensure that the skills of any new professional are an appropriate fit. There is a case for greater support and guidance for practices with general workforce planning and appropriate utilisation, while also recognising that local adaptability and flexibility are key. For GP pharmacists in England, these considerations will be increasingly important in the face of the recent implementation of the structured medication review service, which will undoubtedly lead to renegotiation of existing pharmacist roles and the need to balance the demands of a centrally contracted service with individual practice need.^29^^,^^30^

## Data

The datasets generated and/or analysed during this study are in the form of anonymised interview transcripts. Transcripts are not publicly available but are held on a University of Manchester secure server in line with study ethical approval. Transcripts are available from the corresponding author on reasonable request.
